# Clinical Features of Patients with Dysthymia in a Large Cohort of Han Chinese Women with Recurrent Major Depression

**DOI:** 10.1371/journal.pone.0083490

**Published:** 2013-12-27

**Authors:** Wenqing Wu, Zhoubing Wang, Yan Wei, Guanghua Zhang, Shenxun Shi, Jingfang Gao, Youhui Li, Ming Tao, Kerang Zhang, Xumei Wang, Chengge Gao, Lijun Yang, Kan Li, Jianguo Shi, Gang Wang, Lanfen Liu, Jinbei Zhang, Bo Du, Guoqing Jiang, Jianhua Shen, Ying Liu, Wei Liang, Jing Sun, Jian Hu, Tiebang Liu, Xueyi Wang, Guodong Miao, Huaqing Meng, Yi Li, Chunmei Hu, Yi Li, Guoping Huang, Gongying Li, Baowei Ha, Hong Deng, Qiyi Mei, Hui Zhong, Shugui Gao, Hong Sang, Yutang Zhang, Xiang Fang, Fengyu Yu, Donglin Yang, Tieqiao Liu, Yunchun Chen, Xiaohong Hong, Wenyuan Wu, Guibing Chen, Min Cai, Yan Song, Jiyang Pan, Jicheng Dong, Runde Pan, Wei Zhang, Zhenming Shen, Zhengrong Liu, Danhua Gu, Xiaoping Wang, Xiaojuan Liu, Qiwen Zhang, Yihan Li, Yiping Chen, Kenneth S. Kendler, Jonathan Flint, Zhen Zhang

**Affiliations:** 1 No. 4 Affiliated Hospital of Jiangsu University, Zhenjiang, Jiangsu, P.R. China; 2 Shanghai Mental Health Center, Shanghai, P.R. China; 3 Huashan Hospital of Fudan University, Shanghai, P.R. China; 4 Chinese Traditional Hospital of Zhejiang, Hangzhou, Zhejiang, P.R. China; 5 No. 1 Hospital of Zhengzhou University, Zhengzhou, Henan, P.R. China; 6 Xinhua Hospital of Zhejiang Province, Hangzhou, Zhejiang, P.R. China; 7 No. 1 Hospital of Shanxi Medical University, Taiyuan, Shanxi, P.R. China; 8 ShengJing Hospital of China Medical University, Heping District, Shenyang, Liaoning, P.R. China; 9 No. 1 Hospital of Medical College of Xian Jiaotong University, Xian, Shaanxi, P.R. China; 10 Jilin Brain Hospital, Siping, Jilin, P.R. China; 11 Mental Hospital of Jiangxi Province, Nanchang, Jiangxi, P.R. China; 12 Xian Mental Health Center, New Qujiang District, Xian, Shaanxi, P.R. China; 13 Beijing Anding Hospital of Capital University of Medical Sciences, Deshengmen wai, Xicheng District, Beijing, P.R. China; 14 Shandong Mental Health Center, Jinan, Shandong, P.R. China; 15 No. 3 Hospital of Sun Yat-sen University, Tianhe District, Guangzhou, Guangdong, P.R. China; 16 Hebei Mental Health Center, Baoding, Hebei, P.R. China; 17 Chongqing Mental Health Center, Jiangbei District, Chongqing, P.R. China; 18 Tianjin Anding Hospital, Hexi District, Tianjin, P.R. China; 19 The First Hospital of China Medical University, Heping District, Shenyang, Liaoning, P.R. China; 20 Psychiatric Hospital of Henan Province, Xinxiang, Henan, P.R. China; 21 Nanjing Brain Hospital, Nanjing, Jiangsu, P.R. China; 22 Harbin Medical University, Nangang District, Haerbin, Heilongjiang, P.R. China; 23 Shenzhen Kang Ning Hospital, Luohu District, Shenzhen, Guangdong, P.R. China; 24 First Hospital of Hebei Medical University, Shijiazhuang, Hebei, P.R. China; 25 Guangzhou Brain Hospital (Guangzhou Psychiatric Hospital), Liwan District, Guangzhou, Guangdong, P.R. China; 26 No. 1 Hospital of Chongqing Medical University, Yuanjiagang,Yuzhong District, Chongqing, P.R. China; 27 Dalian No. 7 Hospital, Ganjingzi District, Dalian, Liaoning, P.R. China; 28 No. 3 Hospital of Heilongjiang Province, Beian, Heilongjiang, P.R. China; 29 Wuhan Mental Health Center, Wuhan, Hubei, P.R. China; 30 Sichuan Mental Health Center, Mianyang, Sichuan, P.R. China; 31 Mental Health Institute of Jining Medical College, Dai Zhuang, Bei Jiao, Jining, Shandong, P.R. China; 32 Liaocheng No. 4 Hospital, Liaocheng, Shandong, P.R. China; 33 Mental Health Center of West China Hospital of Sichuan University, Wuhou District, Chengdu, Sichuan, P.R. China; 34 Suzhou Guangji Hospital, Suzhou, Jiangsu, P.R. China; 35 Anhui Mental Health Center, Hefei, Anhui, P.R. China; 36 Ningbo Kang Ning Hospital, Zhenhai District, Ningbo, Zhejiang, P.R. China; 37 Changchun Mental Hospital, Changchun, Jilin, P.R. China; 38 No. 2 Hospital of Lanzhou University, Lanzhou, Gansu, P.R. China; 39 Fuzhou Psychiatric Hospital, Cangshan District, Fuzhou, Fujian, P.R. China; 40 Harbin No. 1 Special Hospital, Haerbin, Heilongjiang, P.R. China; 41 Jining Psychiatric Hospital, North Dai Zhuang, Rencheng District, Jining, Shandong, P.R. China; 42 No. 2 Xiangya Hospital of Zhongnan University, Furong District, Changsha, Hunan, P.R. China; 43 Xijing Hospital of No. 4 Military Medical University, Xian, Shaanxi, P.R. China; 44 Mental Health Center of Shantou University, Shantou, Guangdong, P.R. China; 45 Tongji University Hospital, Shanghai, P.R. China; 46 Huaian No. 3 Hospital, Huaian, Jiangsu, P.R. China; 47 Huzhou No. 3 Hospital, Huzhou, Zhejiang, P.R. China; 48 Mudanjiang Psychiatric Hospital of Heilongjiang Province, Xinglong, Mudanjiang, Heilongjiang, P.R. China; 49 No. 1 Hospital of Jinan University, Guangzhou, Guangdong, P.R. China; 50 Qingdao Mental Health Center, Shibei District, Qingdao, Shandong, P.R. China; 51 Guangxi Longquanshan Hospital, Yufeng District, Liuzhou, P.R. China; 52 Daqing No. 3 Hospital of Heilongjiang Province, Ranghulu District, Daqing, Heilongjiang, P.R. China; 53 Tangshan No. 5 Hospital, Lunan District, Tangshan, Hebei, P.R. China; 54 Anshan Psychiatric Rehabilitation Hospital, Lishan District, Anshan, Liaoning, P.R. China; 55 Weihai Mental Health Center, Qilu Avenue, ETDZ, Weihai, Shandong, P.R. China; 56 Renmin Hospital of Wuhan University, Wuchang District, Wuhan, Hubei, P.R. China; 57 Tianjin First Center Hospital, Hedong District, Tianjin, P.R. China; 58 Hainan Anning Hospital, Haikou, Hainan, P.R. China; 59 Wellcome Trust Centre for Human Genetics, Oxford, United Kingdom; 60 Clinical Trial Service Unit, Richard Doll Building, Oxford, United Kingdom; 61 Virginia Institute for Psychiatric and Behavioral Genetics, Department of Psychiatry, Virginia Commonwealth University, Richmond, Virginia, United States of America; University of Electronic Science and Technology of China, China

## Abstract

**Background:**

Dysthymia is a form of chronic mild depression that has a complex relationship with major depressive disorder (MDD). Here we investigate the role of environmental risk factors, including stressful life events and parenting style, in patients with both MDD and dysthymia. We ask whether these risk factors act in the same way in MDD with and without dysthymia.

**Results:**

We examined the clinical features in 5,950 Han Chinese women with MDD between 30–60 years of age across China. We confirmed earlier results by replicating prior analyses in 3,950 new MDD cases. There were no significant differences between the two data sets. We identified sixteen stressful life events that significantly increase the risk of dysthymia, given the presence of MDD. Low parental warmth, from either mother or father, increases the risk of dysthymia. Highly threatening but short-lived threats (such as rape) are more specific for MDD than dysthymia. While for MDD more severe life events show the largest odds ratio versus controls, this was not seen for cases of MDD with or without dysthymia.

**Conclusions:**

There are increased rates of stressful life events in MDD with dysthymia, but the impact of life events on susceptibility to dysthymia with MDD differs from that seen for MDD alone. The pattern does not fit a simple dose-response relationship, suggesting that there are moderating factors involved in the relationship between environmental precipitants and the onset of dysthymia. It is possible that severe life events in childhood events index a general susceptibility to chronic depression, rather than acting specifically as risk factors for dysthymia.

## Introduction

Dysthymia is a form of chronic depression characterized by depressed mood and symptoms lasting two years or more [1], [2], [3], [4]. Dysthymic patients tend to have a fluctuating course [5] and often have a superimposed episode of major depressive disorder (MDD). This phenomenon has been named “Double Depression” (DD) [4]. Although MDD and dysthymia are highly comorbid, the extent to which the two conditions reflect two separate disease entities is still not fully settled [6], [7], [8], [9]. Some reports indicate that MDD and dysthymia differ in clinical features, progression [3] and family history of mood disorders [10], [11], [12].

One feature thought to differentiate dysthymia from MDD is sensitivity to the environment. Undesirable or stressful life events are a recognized precursor of affective illness, and in a proportion of cases are likely to have an aetiological role (reviewed in [13]). The role that stressful life events play in dysthymia is less well researched [14]. Some reports indicate that stressful life eventsoccur more frequently before the onset of dysthymia, [15], [16]. Chronic uncontrollable stressful life events may be as important in the aetiology of dysthymia as they are for MDD. While some studies show that stressful life events are not a consistent predictor of chronicity in depression [17], it is possible that specific types of stressful life event may more frequently precede the onset of dysthymia than MDD [18], [19]. For example, one possibility is that enduring events, such as the loss of a partner, serious illness or redundancy may be predisposing factors for dysthymia [15], [20], [21]. However the literature on the role of life events in patients with MDD has used small sample sizes (we could not find papers reporting data from more than a 100 cases), and is likely underpowered to detect effects attributable to different categories of life event. Furthermore, we could not find any reports comparing the prevalence and nature of stressful life events in MDD versus controls, with stressful life events in MDD with and without dysthymia. Given that almost all patients with dysthymia develop at least one episode of MDD[9], this comparison is likely to yield useful information about the relative importance of life events in the two disorders.

In this paper we address the issue of the relationship between risk factors for MDD and dysthymia as part of the China, Oxford and VCU Experimental Research on Genetic Epidemiology (CONVERGE) study of MDD. This study, initiated in 2008, has now obtained detailed clinical characteristics and information on known risk factors for MDD in 5,950 cases of recurrent MDD. An initial study published in 2011, reported results from 2,000 cases of recurrent MDD [22]. Here we extend our observations to explore the nature of the relationship between dysthymia and sensitivity to the environment.

## Methods

### Ethics statement

The study protocol was approved centrally by the Ethical Review Board of Oxford University (Oxford Tropical Research Ethics Committee) and the ethics committees in all participating hospitals in China. Major psychotic illness and drug and alcohol abuse were exclusion criteria, and the large majority of patients were seen as out-patients. All patients were judged to be sufficiently in remission to make an informed decision about study participation. Participants provided written consent. All interviewers were mental health professionals who are well able to judge decisional capacity. The study posed minimal risk (an interview and saliva sample).

### Subjects

The data for the present study were drawn from the ongoing China, Oxford and VCU Experimental Research on Genetic Epidemiology (CONVERGE) study. Cases were recruited from 51 provincial mental health centres and psychiatric departments of general medical hospitals in 40 cities in 21 provinces over a five year period from 2008 to 2013. All subjects were female and had four Han Chinese grandparents. Cases were excluded if they had a pre-existing history of bipolar disorder, any type of psychosis or mental retardation. Cases were aged between 30 and 60, had two or more episodes of MD, with the first episode occurring between 14 and 55 and had not abused drug or alcohol before the first episode of MD.

All interviewers were trained by the CONVERGE team for a minimum of one week in the use of the interview. The interview includes assessment of psychopathology, demographic and personal characteristics, and psychosocial functioning. Interviews were tape-recorded and trained editors, who provided feedback on the quality of the interviews, listened to a proportion of them.

### Measures

All subjects were interviewed using a computerised assessment system. The diagnoses of depressive (Dysthymia and Major Depressive Disorder) and anxiety disorders (Generalised Anxiety Disorder, Panic Disorder) were established with the Composite International Diagnostic Interview (CIDI) (WHO lifetime version 2.1; Chinese version), which classifies diagnoses according to the Diagnostic and Statistical Manual of Mental Disorders (DSM-IV) criteria [23].

The interview was originally translated into Mandarin by a team of psychiatrists in Shanghai Mental Health Centre with the translation reviewed and modified by members of the CONVERGE team. Phobias, divided into five subtypes (animal, situational, social and blood-injury, and agoraphobia) were diagnosed using an adaptation of DSM-III criteria requiring one or more unreasonable fears, including fears of different animals, social phobia and agoraphobia that objectively interfered with the respondent's life. The section on the assessment of phobias was translated by the CONVERGE team from the interview used in the Virginia Adult Twin Study of Psychiatric and Substance Use Disorders (VATSPUD)[24]. Neuroticism was measured with the 23-item Eysenck Personality Questionnaire[25]. Information on postnatal depression was assessed using an adaptation of the Edinburgh Scale [26]

We used a stressful life events questionnaire, developed for the VATSPSUD study[24]. The questionnaire assesses 16 traumatic lifetime events and the age of their occurrence. The childhood sexual abuse (CSA) questionnaire was a shortened version of the detailed module used in the VATSPSUD study, which is in turn based on the instrument developed by Martin[27]. Because of evidence that sensitive subjects like CSA are more accurately reported with more confidential methods of assessment [28], subjects were asked to fill in a paper questionnaire about childhood sexual abuse [27]. The questions asked whether, before the subject was 16, did any adult, or any other older person, involve the subject in any unwanted incidents like inviting or requesting them to do something sexual, (2) kissing or hugging in a sexual way, (3) touching or fondling private parts, (4) showing their sex organs, (5) making them touch the person in a sexual way, or (6) attempting or having sexual intercourse. The possible responses were “never,” “once,” and “more than once.” We used these responses to define three forms of CSA [29]: (1) *nongenital* CSA including sexual invitation, sexual kissing, and exposing (2) *genital* CSA including fondling and sexual touching and (3) attempted or completed *intercourse*.

We assessed separately the history of MDD in mothers and father of our cases and controls using the Family History Research Diagnostic criteria [30], and parent-child relationships were measured with the 16 item Parental Bonding Instrument (PBI) modified by Kendler [31] based on Parker's original 25 item instrument[32]. Three factors were extracted from these 16 items and labelled warmth, protectiveness and authoritarianism.[33]. All of the seven original care items from the PBI are included in the 16-item version (items 1, 4, 5, 11, 12, 17, 18 as originally numbered) as well as nine items from the original overprotection scale (items 7, 8, 9, 13, 15, 19, 21, 23, 25).

All subjects interview were fully computerized into a bilingual system of Mandarin and English developed in house in Oxford, and called SysQ. Skip patterns were built into SysQ. Interviews were administered by trained interviewers and entered offline in real time onto SysQ, which was installed on interviewers' laptops. Once an interview was completed, the data, together with an audio recording of the entire interview, was uploaded to a server and transferred to Oxford for analysis.

Additional information was collected using instruments developed and employed in the Virginia Adult Twin Study of Psychiatric and Substance use Disorders [24], translated and reviewed for accuracy by members of the CONVERGE team.

### Statistical analysis

Statistical analyses were performed using the software package SPSS 17.0 (SPSS Inc., Chicago, IL). Scores for neuroticism were standardized to a mean of 0 and a standard deviation of 1. All clinical characteristics, environment sensitivity, patterns of co-morbidity and other continuous variables of individuals with dysthymia vs non-dysthymia MD were assessed by logistic regression in MD, with dysthymia as the dependent variable (0 =  absence and 1 =  presence). Coefficient values, odds ratios and 95% confidence intervals were used to quantify the strength of associations. The statistical significance for all tests was set at *P*<0.05 and corrected where necessary for multiple testing using a Bonferroni correction.

## Results

### Replication

In our sample of 5,950 MDD cases, 587 subjects were diagnosed with dysthymia, a rate of 9.9%. We attempted to replicate the results of the prior analysis of first 2,000 cases [22] by separately performing the same analyses in 3,950 new cases. While sample collection has continued uninterrupted since 2008, we reported data from the first 2,000 cases in 2011[22]. We divided up the two cohorts by date: the first were collected from the beginning of 2008 to the middle of 2010 and the second from mid-2010 to the end of 2012. The results (Table 1) show remarkably consistent findings between the two samples with no significant differences between the two data sets. This consistency justified combining the entire sample, results from which are also provided in table 1. In the rest of this paper, all results are given for the complete sample.

**Table 1:** Relation between dysthymia and clinical features: replication and analysis of complete data set.

Clinical Feature of MDD	Cohort 1 OR	Cohort 2 OR	P-value of difference	COMBINED OR	COMBINED 95% CI	COMBINED P-value	Dysthymia	No Dysthymia
	n = 1,970	n = 3,950					n = 588	n = 5,363
Age of onset	0.96***	0.96***	0.42	0.95	0.94–0.96	5.49E-23	30.98	35.26
Duration of longest episode	1.04***	1.00***	0.34	1.01	1.00–1.01	1.45E-14	72 months	52 months
Number of episodes of MDD	1.03***	1.04***	0.21	1.05	1.04–1.07	4.55E-15	8.6	4.8
Number of stressful life events	1.24***	1.31***	0.45	1.29	1.24–1.35	4.54E-32	2.46	1.46
Family history of MDD	1.27***	1.43***	0.51	1.4	1.25–1.56	7.01E-10	37.10%	24.67%
Neuroticism (standardized values)	1.12***	1.11***	0.27	1.11	1.09–1.13	1.43E-36	1.11	0.61
**Comorbid Diseases**								
Agoraphobia	1.48***	2.08***	0.78	2.19	1.76–2.72	1.61E-12	21.21%	10.96%
Social phobia	1.46***	2.95***	0.4	2.81	2.27–3.49	6.00E-21	22.78%	9.42%
Animal phobia	1.14**	2.01***	0.98	2.06	1.72–2.48	7.63E-15	35.47%	21.04%
Situational phobia	1.36***	2.26***	0.56	2.23	1.83–2.74	5.86E-15	26.08%	13.66%
Blood phobia	1.32***	1.97***	0.96	2.04	1.67–2.49	2.83E-12	26.26%	14.86%
Generalized anxiety disorder	1.87***	2.07***	0.89	2.13	1.78–2.55	7.16E-16	40.20%	24.02%
Melancholia	0.93	0.87	0.68	1.05	0.82–1.35	0.68	86.54%	85.92%
Postnatal Depression	2.05***	1.74***	0.07	2.06	1.68–2.52	2.62E-12	29.69%	16.91%
Panic	2.71***	2.06***	0.6	2.28	1.74–2.98	1.81E-09	12.08%	6.07%

The table shows results from two cohorts as well as the combined dataset. Cohort 1 was collected from the beginning of 2008 to the middle of 2010 and cohort 2 from mid 2010 to the end of 2012. The last two columns show the means, or where appropriate the percentages (%), of each clinical feature for the combined data set. ‘Dysthymia’ refers to MDD cases with dysthymia and ‘No dysthymia’ refers to MDD without dysthymia. Abbreviations used: MDD, major depressive disorer; OR: odds ratio. 95% CI: 95% confidence intervals. *p<0.05 **p<0.01 ***p<0.001

Table 1 confirms that, compared to those with MDD alone, those with dysthymia suffer slightly more episodes of MDD, have longer episodes, higher rates of comorbid anxiety disorders, higher levels of neuroticism, are more likely to have a family history of MDD and have experienced more stressful life events.

### Sensitivity to the environment

We investigated the relationship between susceptibility to dysthymia and environmental risk factors for depression. We attempted to determine if some life events specifically predispose to dysthymia in those patients with MDD compared to those with MDD alone. In order to do so, we chose only those life events that were reported to precede the first onset of dysthymia. Our comparison group is those with MDD without dysthymia. We assigned an artefactual age of onset to this group, using the known distribution of age of onset for dysthymia so as to apply an equivalent risk of exposure in the two groups. We then assessed the association of each life event with dysthymia (using logistic regression) and show the results in Table 2.

**Table 2:** Relation between dysthymia with MDD and stressful life events.

Life Event	P-value	OR	95%CI	Dysthymia	No dysthymia
				n = 588	n = 5363
Death of a family member	0.736	1.06	0.77 – 1.46	21.09%	19.54%
Divorce/relationship breakup	0.0042	1.57	1.15 – 2.13	30.61%	16.65%
Ever unemployed	0.0171	1.45	1.07 – 1.96	23.81%	14.58%
Job loss	0.274	1.3	0.81 – 2.06	10.03%	6.97%
Financial crisis	0.00123*	1.54	1.19 – 2.01	31.46%	17.75%
Legal problems	0.000905*	2.67	1.50 – 4.78	7.48%	3.24%
Serious illness	0.787	1.05	0.72 – 1.54	14.97%	11.26%
Life-threatening accident	0.106	1.36	0.94 – 1.97	10.37%	7.76%
Natural disaster	0.0173	1.44	1.07 – 1.94	12.41%	9.85%
Witness someone injured	0.365	1.2	0.81 – 1.79	10.20%	7.55%
Raped	0.0161	2.05	1.14 – 3.68	3.74%	1.60%
Physically assaulted	6.12E-06***	2.21	1.57 – 3.11	12.93%	6.17%
Physically abused as a child	1.50E-12***	3.1	2.27 – 4.25	10.03%	3.62%
Seriously neglected as a child	6.63E-12***	2.29	1.81 – 2.90	18.03%	8.91%
Threatened	0.0017	2.84	1.48 – 5.46	3.23%	1.29%
Other terrible event	0.117	1.34	0.93 – 1.93	12.24%	7.35%
Childhood sexual abuse					
Non genital	1.58489E-15***	2.63	2.07 – 3.33	6.12%	2.83%
Genital	3.98107E-10***	2.38	1.81 – 3.12	8.16%	3.69%
Intercourse	0.000316228**	2.12	1.41 – 3.18	5.10%	2.50%

The table shows the result of assessing the relationship between dysthymia and life events that were reported to precede the first onset of dysthymia, within cases of major depression. The table gives the P-value, odds ratio and 95% confidence intervals (95% CI) for logistic regression and sixteen stressful life events, and childhood sexual abuse (reported as either non genital, genital or intercourse). The last two columns show the percentage of cases reporting each event, regardless of the age of onset. ‘Dysthymia’ refers to MDD cases with dysthymia and ‘No dysthymia’ refers to MDD without dysthymia. The thresholds for P-values corrected for multiple testing are shown as *p<0.05 **p<0.01 ***p<0.001.

**Figure 1:**
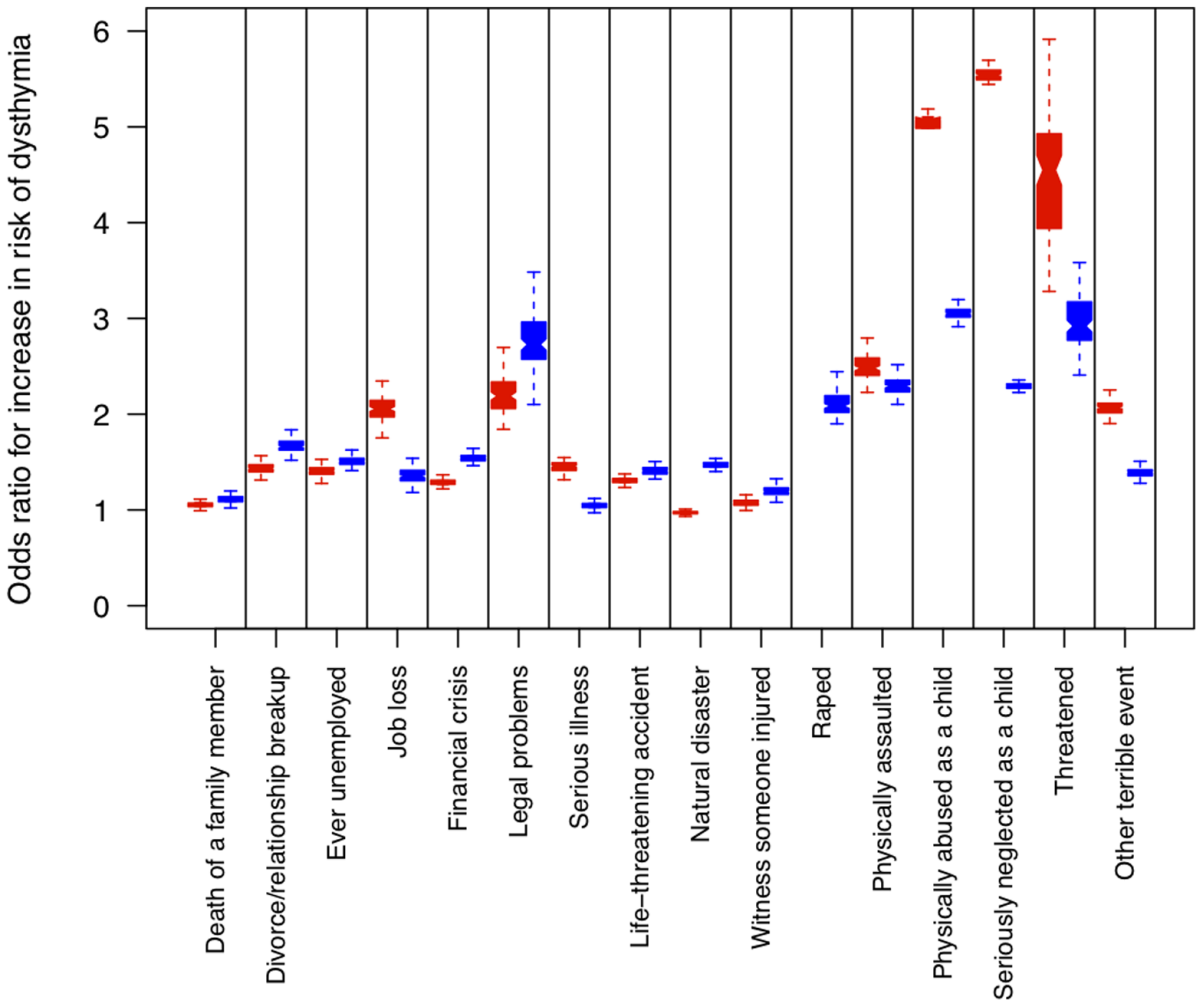
Distribution of odds ratios for events preceding the onset of dysthymia and major depressive disorder. The vertical axis shows the odds ratios, the horizontal axis the sixteen stressful life events that precede the onset of MDD alone (shown as a red box plot) and dysthymia with MDD (blue boxes). Box plots show the distribution of comparisons with 100 simulated ages of onset. Note that the result for the rape event on MDD alone is not shown because the OR is 40.

Ten of the sixteen stressful life events we assessed were significantly more likely (at an uncorrected threshold of 5%) to precede the onset of disease in patients with dysthymia and MDD, compared to patients with MDD alone (Table 2). After applying a multiple testing correction, we observed that five stressful life events were significantly associated. We also found that the effect of childhood sexual abuse (CSA) was significant at a corrected 5% level. However a single assignment of the artefactual age of onset might not provide an accurate estimate, so to determine the robustness of the results, we repeated the analysis 100 times, each time randomly assigning a new artefactual age of onset in the MDD only group. Figure 1 shows the distribution of effects for the sixteen stressful life events, comparing dysthymia given MDD (blue boxes), with MDD alone (red boxes).

Table 2 and Figure 1 indicate that the pattern of effects of stressful life events on MDD alone and MDD with dysthymia differs in three ways. First, the relative severity of life events correlates with the odds ratio observed for MDD, but less so for dysthymia and MDD. For example the more severe forms of CSA (intercourse and genital contact) have lower odds ratios for dysthymia than the least severe (non-genital contact) (Table 2). By contrast, in MDD increasing severity of CSA is associated with an increasing odds ratio. Second, there is an enormous difference for the effect of rape. The mean OR for MDD alone is 33.1 compared to 2.1 for MDD with dysthymia. Third, the stressful life events with a higher OR for dysthymia shown in Figure 1 include those of a more chronic nature: the loss of a close relative or partner, being unemployed, financial and legal difficulties. Overall this pattern suggests that highly threatening but short-lived threats (such as rape) are less likely to predispose to dysthymia in patients with MDD than more chronically stressful events.

Finally we examined the contribution of perceived parenting to the susceptibility to dysthymia. For this analysis, we used the Parental Bonding Instrument from which we extracted three factors: warmth, protectiveness and authoritarianism. High warmth reflected a loving parenting style, high protectiveness an overprotective and controlling parental style, and highly authoritarian parenting discourages a child's sense of independence and autonomy. Our results, in table 3, show that low parental warmth, from either mother or father, increases the risk of dysthymia. We also observed an effect of a perceived authoritarianism in the father (but not in the mother). Protectiveness slightly, but non-significantly (after applying a correction for multiple testing), increases the risk of dysthymia.

**Table 3:** Contribution of perceived parenting to the susceptibility to dysthymia.

Perceived parenting	OR	95%CI	*P-value*
Maternal warmth	0.94	0.93– 0.96	4.51E-10
Paternal warmth	0.94	0.92– 0.96	1.34E-10
Maternal authoritarianism	1.04	1.01 – 1.06	0.014
Paternal authoritarianism	1.05	1.02 – 1.08	0.0003
Maternal protectiveness	1.04	1.01– 1.08	0.0024
Paternal protectiveness	1.03	0.99 – 1.07	0.018

The table shows the P-value, odds ratio and 95% confidence intervals (95% CI) for the relationship between dysthymia and three facets of perceived parenting. Results are reported for both maternal and paternal perceived parenting values.

## Discussion

Our investigation into the clinical characteristics of dysthymia has made two important observations. First, we were able to replicate findings in a large sample, speaking to the robustness of the diagnoses and information we have collected in this very large sample of women with recurrent MDD. Second, we find that the relationship between stressful life events and dysthymia in the presence of MDD is not the same as that between stressful life events and MDD. While we find increased odds ratios for all stressful life events, consistent with their role as aetiological factors, we do not so clearly observe a dose response relationship, with the more severe events showing the larger odds ratio. Instead, as is shown for results for childhood sexual abuse, the more severe forms are actually associated with a lower odds ratio.

Our results add to a literature that attempts to determine the diagnostic status of dysthymia. Consistent with the origin of dysthymia in DSM-III with the alternative title of “depressive neurosis”, our sample has higher levels of neuroticism and comorbidity with anxiety disorders. MDD patients with dysthymia are known to have greater comorbidity [3]. As others have done, we also observed increased rates of a family history of MD [10], [11], [12].

We observed a complex relationship between dysthymia and known environmental risk factors for MDD. While there are clearly increased rates of stressful life events preceding the onset of dysthymia in patients with MDD, the impact of life events on susceptibility to dysthymia with MDD differs from that of patients with MDD alone. We found some evidence that high threat but short-lived events (such as rape) were more specific for MDD, while stressful life events impacting on dysthymia were enriched for events that occur early in life and are likely to have persistent, long lasting consequences (such as neglect and abuse). This is consistent with reports that enduring events may be predisposing factors for dysthymia [15], [20], [21].

However, for dysthymia, in contrast to the pattern seen in MDD, the more severe forms of childhood sexual abuse (intercourse and genital contact) had lower odds ratios than the least severe (non-genital contact), arguing against a simple dose-response relationship. This suggests that there may be moderating factors involved in the relationship between CSA and the onset of dysthymia. Childhood events may index a general susceptibility to chronic depression. In this context it may be relevant that a lack of parental warmth and paternal authoritarianism increase the risk for dysthymia over and above a risk for MDD. There may be features of upbringing and childhood experience that predispose to dysthymia in later life, possibly rendering subjects more liable to the effects of protracted but low threat events that we found to be enriched in cases with MDD and dysthymia.

A number of limitations should be born in mind in the interpretation of our results. First, our data were acquired retrospectively and on a single occasion, thus potentially biasing our assessment of environmental factors, particularly stressful life events. Second, our results were acquired in a patients ascertained through hospitals and we cannot know whether they extrapolate to community acquired samples. Third, our sample consists of Chinese women with recurrent MDD. Our results may not apply to men with depression or to those who suffer single episode.
